# Hepcidin-25 in Diabetic Chronic Kidney Disease Is Predictive for Mortality and Progression to End Stage Renal Disease

**DOI:** 10.1371/journal.pone.0123072

**Published:** 2015-04-20

**Authors:** Martin Wagner, Damien R. Ashby, Caroline Kurtz, Ahsan Alam, Mark Busbridge, Ulrike Raff, Josef Zimmermann, Peter U. Heuschmann, Christoph Wanner, Lothar Schramm

**Affiliations:** 1 Division of Nephrology, Department of Medicine I, University Hospital Würzburg, Würzburg, Germany; 2 Institute of Clinical Epidemiology and Biometry, University of Würzburg, Würzburg, Germany; 3 Comprehensive Heart Failure Center, University of Würzburg, Würzburg, Germany; 4 Kidney and Transplant Institute, Imperial College, London, United Kingdom; 5 Division of Nephrology, McGill University, Montreal, Canada; 6 Department of Clinical Chemistry, Imperial College, London, United Kingdom; 7 Division of Nephrology and Hypertension, Department of Internal Medicine 4, University of Erlangen-Nürnberg, Erlangen, Germany; 8 Clinical Trial Unit, University Hospital Würzburg, Würzburg, Germany; Children's Hospital Boston/Harvard Medical School, UNITED STATES

## Abstract

**Background:**

Anemia is common and is associated with impaired clinical outcomes in diabetic chronic kidney disease (CKD). It may be explained by reduced erythropoietin (EPO) synthesis, but recent data suggest that EPO-resistance and diminished iron availability due to inflammation contribute significantly. In this cohort study, we evaluated the impact of hepcidin-25—the key hormone of iron-metabolism—on clinical outcomes in diabetic patients with CKD along with endogenous EPO levels.

**Methods:**

249 diabetic patients with CKD of any stage, excluding end-stage renal disease (ESRD), were enrolled (2003–2005), if they were not on EPO-stimulating agent and iron therapy. Hepcidin-25 levels were measured by radioimmunoassay. The association of hepcidin-25 at baseline with clinical variables was investigated using linear regression models. All-cause mortality and a composite endpoint of CKD progression (ESRD or doubling of serum creatinine) were analyzed by Cox proportional hazards models.

**Results:**

Patients (age 67 yrs, 53% male, GFR 51 ml/min, hemoglobin 131 g/L, EPO 13.5 U/L, hepcidin-25 62.0 ng/ml) were followed for a median time of 4.2 yrs. Forty-nine patients died (19.7%) and forty (16.1%) patients reached the composite endpoint. Elevated hepcidin levels were independently associated with higher ferritin-levels, lower EPO-levels and impaired kidney function (all p<0.05). Hepcidin was related to mortality, along with its interaction with EPO, older age, greater proteinuria and elevated CRP (all p<0.05). Hepcidin was also predictive for progression of CKD, aside from baseline GFR, proteinuria, low albumin- and hemoglobin-levels and a history of CVD (all p<0.05).

**Conclusions:**

We found hepcidin-25 to be associated with EPO and impaired kidney function in diabetic CKD. Elevated hepcidin-25 and EPO-levels were independent predictors of mortality, while hepcidin-25 was also predictive for progression of CKD. Both hepcidin-25 and EPO may represent important prognostic factors of clinical outcome and have the potential to further define “high risk” populations in CKD.

## Introduction

Anemia is common in patients with chronic kidney disease (CKD) and diabetes and is related to worse prognosis [1–3]. Herein, anemia enhances well-known diabetic microvascular complications, while a variety of molecular pathways have been identified [4]. Microvascular and macrovascular complications of diabetes can be explained by anemia further deteriorating tissue hypoxia [5], which is the main stimulus of endogenous erythropoietin (EPO) release [6].

EPO is the most important hormone of hemoglobin regulation and reduced production of EPO (“absolute EPO deficiency”) might be a major cause of decreasing hemoglobin levels in CKD [7]. However, anemia can frequently be detected even in early stages of diabetic CKD [8] as well as in a multitude of other chronic diseases (“anemia of chronic diseases” [ACD]) [5, 9]. Processes of chronic low-grade inflammation are characteristic for these conditions and may also be causal for impairments of hemoglobin synthesis [5, 10]. In ACD and anemia of CKD, alterations of EPO-related mechanisms are discussed, e.g. “relative EPO deficiency”, that is inappropriately low levels of EPO (however, within a “normal range” in a non-anemic reference population) despite low hemoglobin levels. This scenario could indicate either sensing errors and/or insufficient synthesis of EPO. In contrast, elevated EPO-levels have also been described in anemic patients; a phenomenon that could be explained by resistance of the bone marrow to EPO [11–13].

Dysregulation of iron homeostasis represents another key-player in ACD; levels of iron in the circulation are decreased as intestinal iron-absorption is reduced and the release of storage iron is inhibited [5, 14]. The hormone hepcidin, with its active isoform hepcidin-25, seems to be the main regulator of iron homeostasis in this setting [15] and is itself regulated by inflammatory processes [16, 17].

While anemia and chronic inflammation are frequently detected in CKD, hepcidin-25 is an important biomarker, determining impaired iron metabolism in ACD [18, 19]. However, evidence on the prognostic implications of hepcidin-25 is sparse, as only few reports described its association with clinical outcome [20–22], and importantly, to our knowledge, not by considering endogenous EPO levels simultaneously.

Previously, we have shown that in a group of patients with type 2 diabetic CKD, elevated EPO levels were strongly associated with classical markers of inflammation and were also independently predictive for mortality [23]. The purpose of the current study was to investigate hepcidin-25 levels in the setting of diabetic patients with CKD with a focus on its association with EPO levels and other variables of CKD, anemia and inflammation. Furthermore, we analyzed its relationship along with EPO and other known risk factors, to mortality and progression of CKD.

## Materials and Methods

As described previously [23], a cohort of 243 adult patients with type 2 diabetes of any CKD stage was enrolled between 2003 and 2005 from four nephrology outpatient clinics in the Würzburg area, Germany. Main exclusion criteria comprised renal replacement therapy (RRT, dialysis or kidney transplantation) at baseline, and any type of anemia therapy (red blood cell transfusions within three weeks before enrollment, medical therapy with iron, vitamin B12, folate, or erythropoietin stimulating agents [ESA]). Information was collected on medical history, physical examination and routine clinical measures, while details on medical history were based on personal interview as well as by detailed investigation of the patients’ charts. Biomaterials were processed immediately and stored at -80°C. Between 2008 and 2009, patients were followed by telephone interview with their nephrologist and/or primary care physician (PCP) regarding survival status, initiation of RRT and the patient’s last available serum creatinine measurement. The latter value was determined prior to death (however, in a considerably stable condition as judged by the patient’s PCP/nephrologist) or most closely to the date of the telephone interview. During the same time of baseline examination, a group of n = 29 type 1 diabetic patients was enrolled in the study according to the procedures described above, while longitudinal information was collected up to 2012. The study was approved by the Ethics Committee of the University of Würzburg. All patients provided written informed consent.

Glomerular filtration rate (GFR) was calculated by taking the average of measured creatinine and urea clearance in a 24-hour urine collection, adjusted for body-surface area [24]. If 24-hour urine collection was missing (5% of the total cohort), GFR was estimated according to the CKD-EPI formula [25]. EPO was measured by ELISA (Roche). For hepcidin measurement, serum samples were thawed and aliquots were filled into tubes at +4°C and frosted again at -80°C immediately thereafter. Samples were shipped on dry ice to the Imperial College, London, UK within 24 hrs. Hepcidin-25 was measured by RIA as previously reported [26] (range of measurement: 1.25 to 160 ng/ml, detection limit: 0.6 ng/ml). As biomaterials were not available for all patients, we studied a total of n = 249 patients (n = 224 type 2, n = 25 type 1 diabetes). Patients with missing serum samples (n = 23) had lower median (inter quartile range, IQR) GFR of 28.5 ml/min (17.7–57.0; p = 0.01) and tended to have a greater proportion of history of cardiovascular disease (CVD, 52.2%, p = 0.07) as compared to patients in whom samples were available (GFR 50.8 ml/min [29.5–70.9]; CVD 32.1%). Other characteristics, such as age, gender, diabetes type, EPO, CRP or hemoglobin did not differ (all p>0.3). Longitudinal outcomes of interest included all-cause mortality and a composite endpoint of *progression of CKD*, defined as either initiation of RRT or doubling of serum creatinine (SCr) in patients not on RRT.

### Statistical Methods

Analyses were performed on a complete case dataset using SAS 9.3 (SAS Institute, Cary, NC, USA). Characteristics of participants were compared across hepcidin tertiles (<44, 44–76, >76 ng/ml) using ANOVA, Kruskal-Wallis test, χ^2^- test, and Fisher’s exact test, as appropriate. Factors associated with hepcidin levels at baseline were examined using linear regression analysis; if needed, variables were transformed to assure correct regression analyses, e.g. the logarithmic form of hepcidin was used. In stepwise forward multivariate analyses, models of basic patient characteristics, renal function and anemia (model 1), and additional markers of inflammation and clinical variables (model 2) were built. The association of hepcidin at baseline with mortality and *progression of CKD* was investigated by Kaplan-Meier analyses across tertiles. In Cox proportional hazards analyses, we investigated the association of hepcidin with clinical endpoints in univariate and in backward multivariate analyses (model 1: p_exclusion_>0.10, model 2: p_exclusion_>0.05), accounting for EPO, the interaction of hepcidin and EPO and other potential confounders/mediators such as age, albumin, and kidney function, in particular those factors that indicated a relationship to hepcidin-25 in linear regression analysis. The functional form of the variables in the Cox models and the proportional hazards assumptions were tested by Schoenfeld residuals. The model’s predictive ability was assessed by time-dependent C-statistics, which describe the probability that the model will assign the higher risk to the patient who achieved the endpoint as compared to the patient who did not.

To test the robustness of results, sensitivity analyses in the final regression models were performed; (a) other kidney function measures: eGFR_CKD-EPI_ for all patients, eGFR_MDRD_ for all patients, eGFR_MDRD_ in those with missing 24hr urine collection; (b) gender, hemoglobin and diabetes-type forced in the analyses, as well as testing the models in type 2 diabetics only; (c) forcing ferritin in the Cox models; (d) excluding patients with iron-deficiency (i.e. ferritin <30 μg/l [27]); (e) excluding patients with CKD stages 1 and 2 and (f) imputing missing data (S1 Methods for imputing missing data).

## Results

Participants were on average 66.5 years old, 53% were male, with a GFR of 51 ml/min (Table 1). Patients in the higher hepcidin tertiles were more likely to be male and in advanced stages of CKD. They were also more likely to be anemic, and with a history of hypertension and hyperlipidemia. EPO-levels were highest in the low hepcidin tertile. CRP-levels did not differ across tertiles as did not hemoglobin or the type of diabetes.

**Table 1:** Patient characteristics and outcomes, total cohort and by hepcidin tertile.

		hepcidin tertile			
	total cohort	<44 ng/ml	44–76 ng/ml	>76 ng/ml	
	n = 249	n = 83	n = 84	n = 82	p-value
age, yrs	66.5 (57.0–73.2)	67.6 (57.2–73.2)	66.0 (55.4–72.5)	66.3 (58.1–74.2)	0.9
gender, male	52.6%	42.2%	52.4%	63.4%	**0.02**
body mass index, kg/m^2^	29.4 (27.0–33.2)	29.3 (26.8–34.1)	29.6 (27.8–32.9)	29.4 (26.4–32.9)	0.8
Diabetes mellitus					0.5
Type 1	10.0%	12.1%	7.1%	11.0%	
Type 2	90.0%	88.9%	92.9%	89.0%	
duration of diabetes, yrs	10 (4–21.5)	10 (3–19)	12 (4–24)	10 (4–22)	0.5
diabetic retinopathy ^a^	32.1%	28.9%	33.3%	34.2%	0.7
history of CVD ^b^	32.1%	37.4%	32.1%	26.8%	0.4
smoking ^c^	27.4%	24.4%	33.3%	24.4%	0.3
hypertension ^a^	81.9%	73.5%	85.7%	86.6%	**0.049**
blood pressure, mmHg					
systolic	143 ± 22	142 ± 20	143 ± 23	144 ± 22	0.9
diastolic	81 ± 14	80 ± 15	81 ± 14	80 ± 12	0.8
hyperlipidemia ^a^	42.2%	32.5%	42.9%	51.2%	**0.052**
**Laboratory**					
GFR, ml/min/1.73m^2^	51 (30–71)	53 (39–76)	51 (31–70)	44 (25–70)	**0.07**
CKD ^d^					**0.05**
stage G1	6.3%	6.6%	4.9%	7.4%	
stage G2	30.5%	29.0%	35.4%	27.2%	
stage G3a	18.0%	25.0%	15.9%	13.6%	
stage G3b	18.8%	23.7%	19.5%	13.6%	
stage G4	19.7%	13.2%	20.7%	24.7%	
stage G5	6.7%	2.6%	3.7%	13.6%	
proteinuria, mg/day	186 (107–1145)	156 (94–530)	182 (119–879)	300 (127–1605)	0.10
HbA1c, %	6.9 (6.4–8.0)	6.9 (6.4–7.8)	7.1 (6.4–8.1)	6.9 (6.4–8.1)	0.9
C-reactive protein, mg/dL	0.34 (0.14–0.78)	0.34 (0.16–0.63)	0.25 (0.11–0.90)	0.37 (0.19–0.99)	0.4
albumin, g/dL	4.1 (3.8–4.4)	4.1 (3.8–4.4)	4.1 (3.9–4.4)	4.2 (3.8–4.6)	0.8
total cholesterol, mg/dL	198 (176–223)	198 (176–221)	194 (174–209)	199 (177–227)	0.6
hemoglobin, g/L	131 ± 20	132 ± 19	133 ± 20	129 ± 19	0.3
anemia ^e^	37.9%	31.1%	33.3%	49.4%	**0.04**
ferritin, μg/L	149 (71–244)	65 (38–112)	154 (105–223)	260 (159–363)	**<0.001**
EPO, U/L	13.5 (9.2–18.4)	15.2 (9.6–23.7)	12.8 (9.1–16.6)	13.4 (9.3–16.7)	**0.04**
hepcidin, ng/ml	62.0 (33.0–83.0)	26 (16.8–33)	62 (55–69)	96 (83–120)	**<0.001**
**Outcomes**					
death	19.7%	22.9%	13.1%	23.2%	0.18
initiation of RRT	14.1%	4.8%	15.5%	22.0%	0.006
doubling of SCr	2.0%	2.6%	2.9%	1.6%	0.9
progression of CKD (RRT or doubling of SCr)	16.1%	7.4%	18.1%	23.2%	0.02

**Legend:** data are means ± standard deviation, medians (interquartile range) and proportions (%); p-value across hepcidin tertiles; abbreviations: EPO, endogenous erythropoietin; CVD, cardiovascular disease, GFR, glomerular filtration rate; TIA, transient ischemic attack; SCr, serum creatinine; RRT, renal replacement therapy.

^a^ self-reported history or as specified in the patient’s chart

^b^ self-reported history of angina pectoris, myocardial infarction, stroke /TIA, or as specified in the patient’s chart

^c^ current smoker or stopped within the past 5 yrs

^d^ CKD stages G1 and G2 defined as proteinuria and GFR >90 ml/min and 60–90 ml/min, respectively

^e^ hemoglobin <120 g/L in women and <135 g/L in men

Information on at least one subsequent clinic appointment after the baseline visit was collected in all patients after a median observation time of 4.2 years (max. 8.6 years). During follow-up, 49 patients died, of which 20 were on RRT. Main causes of death were vascular, e.g. myocardial infarction, stroke, sudden death (n = 19, 38.8%) and due to sepsis/infection (n = 11, 22.5%). The remaining causes were malignancy (n = 5, 10.2%), other (n = 7, 14.3%), and unknown (n = 7, 14.3%). Thirty-five patients progressed to ESRD (34 hemodialysis, 1 peritoneal dialysis) and in those not on dialysis, SCr doubled in five patients. The composite endpoint *progression of CKD* was observed in 40 patients.

### Determinants of Hepcidin-25 levels

In univariate linear regression analyses at baseline, lower hepcidin levels were found in patients with preserved and mildly impaired GFR and those with higher EPO levels (Table 2). Hepcidin-levels were elevated more frequently in male patients and those with hypertension and hyperlipidemia, and those with greater values of proteinuria. Hemoglobin was not associated with hepcidin. In multivariate analyses, the associations of EPO and GFR with hepcidin remained statistically significant, even after adjustment for markers of inflammation and clinical variables, e.g. hypertension and hyperlipidemia (models 1 and 2). Levels of hepcidin and ferritin were strongly correlated, but not entirely co-linear (as assessed by condition indices [28] and variance inflation factors [29]), thus explaining a large amount of variability in hepcidin levels (R^2^ = 0.5).

**Table 2:** Determinants of log-hepcidin-level (linear regression analyses).

	univariate	multivariate	
		model 1	model 2
**age** [10 yrs]	-0.02 (-0.09; 0.05)	0.002, p = 0.5	--
**gender, male**	**0.24 (0.08; 0.41)**	-0.11, p = 0.13	**0.21 (0.04; 0.37)**
**type 2 diabetes**	-0.09 (-0.37; 0.19)	-0.03, p = 0.7	--
**EPO** [log (U/L)]	**-0.02 (-0.39; -0.09)**	-0.007 (-0.04; -0.01), p = 0.065	**-0.01 (-0.02; 0.00)**
**GFR** [10 ml/min/1.73 m^2^]	**-0.04 (-0.07; -0.004)**	**-0.04 (-0.06; -0.01)**	**-0.03 (-0.06; -0.01)**
**proteinuria** [log(mg/day)]	**0.05 (-0.007; 0.11)**	0.02, p = 0.4	--
**hemoglobin** [g/L]	-0.005 (-0.05; 0.04)	-0.01, p = 0.8	--
**ferritin** [log(μg/L)]	**0.48 (0.41; 0.55)**	**0.46 (0.39; 0.53)**	**0.45 (0.38; 0.52)**
**CRP** [log(mg/dl)]	0.03 (-0.03; 0.10)	**--**	0.02, p = 0.6
**albumin** [(g/dL)^2^]	0.002 (-0.02; 0.02)	**--**	-0.002, p = 0.7
**history of CVD**	-0.13 (-0.31; 0.05)	**--**	-0.05, p = 0.5
**hypertension**	**0.19 (-0.02; 0.41)**	**--**	**0.18 (0.02; 0.35)**
**hyperlipidemia**	**0.21 (0.05; 0.39)**	**--**	**0.13 (0.00; 0.25)**

**Legend:** data are beta-coefficients (95% CI), displayed bolded if p<0.05; multivariate linear regression models were built stepwise (model 1: basic patient characteristics, model 2: additional markers of inflammation and clinical variables), while eliminating variables with p>0.1 within each model (beta-coefficients and p-values are displayed before variables left the model); abbreviations: CI, confidence interval; EPO, endogenous erythropoietin; CVD, cardiovascular disease; GFR, glomerular filtration rate; CRP, C-reactive protein;

These findings were not altered in sensitivity analyses testing various GFR estimations, and when gender, hemoglobin and diabetes-type were forced in the model. Similarly, no substantial changes were observed when only type 2 diabetics were investigated or iron deficient patients (n = 14) were excluded. Focusing on advanced stages of CKD (stages 3–5, n = 130) did not change the associations of GFR, EPO and ferritin with hepcidin levels, whereas hypertension and dyslipidemia lost significance (detailed data not shown). Finally, the beta-coefficients of the complete case model 2 were within the confidence limits of the regression model on the imputed dataset (S1 Table. Multivariate linear regression analysis on imputed dataset, dependent variable log-hepcidin).

### Association of Hepcidin-25 with Mortality

**Fig 1:**
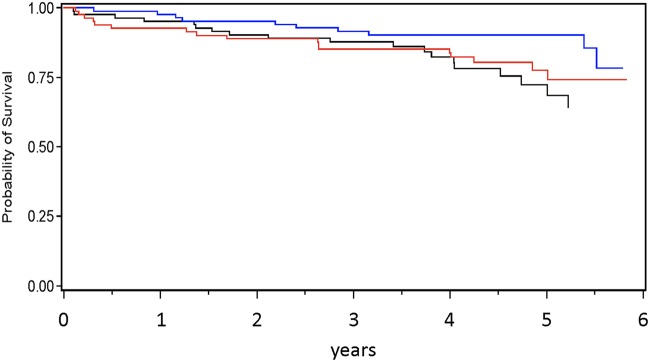
Probability of survival according to hepcidin tertiles. low (black), intermediate (blue), high (red); (Kaplan-Meier analysis, p_log-rank_ = 0.19).

In univariate analyses, no clear relationship between hepcidin and the probability of survival was observed (Fig 1 and Table 3). Older age, male gender, a history of CVD, impaired kidney function (GFR and proteinuria) as well as lower hemoglobin and albumin levels and higher levels of CRP were significantly associated with mortality in univariate analyses. Patients with type 2 diabetes were at higher risk for mortality when compared to type 1 diabetics. In multivariate analyses, male gender, type 2 diabetes, a history of CVD, hemoglobin, albumin and GFR lost significance, while older age, the extent of proteinuria and higher CRP-levels remained significantly related to mortality (all p<0.05). These analyses also revealed an independent association of higher hepcidin levels with a higher risk for mortality, in particular when the interaction of hepcidin and EPO levels was considered simultaneously. Although higher hepcidin levels were (HR_hepcidin_ = 1.49, p = 0.01) and higher EPO levels tended to be (HR_logEPO_ = 2.47, p = 0.08) related to mortality, the interaction of both (HR_hepcidin*logEPO_ = 0.86, p = 0.01) attenuated the risk for mortality. These observations get supported by an increased C-statistic (0.823 ± 0.031), if both hepcidin and EPO were considered in model 2 as compared to the model without these variables (0.809 ± 0.029); while the difference between these C-statistics was not statistically significant (p = 0.4).

**Table 3:** Determinants of mortality (Cox proportional hazards analysis)

	univariate	multivariate	
		model 1	model 2
hepcidin [10 ng/ml]	1.024 (0.951; 1.102)	**1.513 (1.136; 2.015)**	**1.491 (1.095; 2.029)**
EPO [log (U/L)]	1.497 (0.867; 2.585)	2.612 (0.972; 7.020)	2.466 (0.884; 6.877)
hepcidin * logEPO	*--*	***0*.*846 (0*.*759; 0*.*944)***	***0*.*858 (0*.*763; 0*.*9763)***
age [10 yrs]	**1.917 (1.413; 2.602)**	**2.225 (1.399; 3.538)**	**1.982 (1.292; 3.042)**
gender, male	**1.982 (1.089; 3.601)**	2.030 (0.873; 4.721)	2.03, p = 0.10
type 2 diabetes	**3.715 (1.015; 13.60)**	0.78, p = 0.8	--
GFR [10 ml/min/173m^2^]	**0.718 (0.624; 0.826)**	0.96, p = 0.7	--
proteinuria [log(mg/day)]	**1.487 (1.229; 1.799)**	**1.538 (1.227; 1.929)**	**1.625 (1.305; 2.023)**
hemoglobin [g/L]	**0.829 (0.725; 0.949)**	0.99, p = 0.9	--
CRP [log (mg/dl)]	**1.509 (1.194; 1.908)**	**1.592 (1.163; 2.179)**	**1.566 (1.133; 2.165)**
albumin [(g/dl)]	**0.425 (0.276; 0.655)**	0.74, p = 0.5	--
history of CVD	**3.663 (2.041; 6.571)**	1.63, p = 0.2	**--**
hypertension	1.216 (0.566; 2.611)	--	--
hyperlipidemia	1.055 (0.598; 1.864)	--	--
ferritin [log(μg/L)]	0.973 (0.715; 1.324)	--	--

**Legend:** data are hazard ratios (HR) (95% confidence interval, CI), displayed bolded if p<0.05; multivariate Cox models were built with backwards selection (model 1: p_exclusion_>0.10, model 2: p_exclusion_>0.05), accounting for variables significant (p<0.05) in univariate associations (HRs and p-values are displayed before variables left the model)

Ferritin was not associated with mortality, neither in univariate analyses (HR_logferritin_ 0.97, p = 0.9), nor when being forced in the final Cox model 2 (HR_logferritin_ 1.00, p = 0.9), while the relations of the other variables remained stable. In further sensitivity analyses (see methods) the direction as well as magnitude of the associations remained largely unchanged. Restricting the dataset to patients with advanced stages of CKD (i.e. stages 3–5, n = 130 patients, n = 41 outcomes), did not markedly alter the association of hepcidin to mortality and also not the interaction of EPO and hepcidin, but age, gender and history of CVD lost significance (detailed data not shown). Hazard Ratios of the complete-case model were within the 95% confidence limits of hazard ratios derived from the imputation (S2 Table. Multivariate Cox proportional hazards analysis on imputed dataset, outcome mortality).

### Association of Hepcidin-25 with Progression of CKD

**Fig 2:**
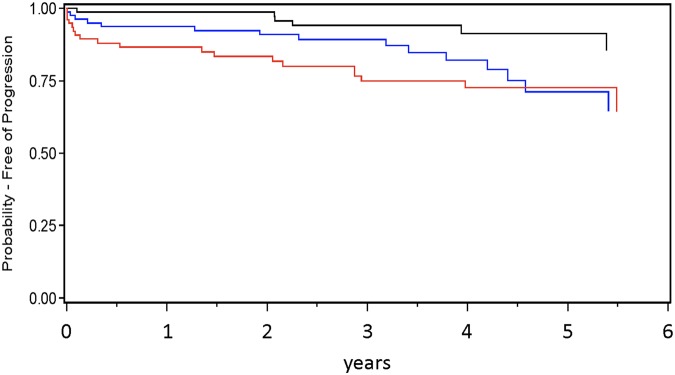
Probability of time free of progression of CKD according to hepcidin tertiles. low (black), intermediate (blue), high (red); (Kaplan-Meier analysis, p_log-rank_ = 0.01).

Elevated levels of hepcidin were related to a higher risk for *progression of CKD* (Fig 2 and Table 4). Moreover, older age, male gender, a history of CVD, higher levels of CRP and proteinuria, and lower levels of albumin, hemoglobin and baseline GFR were associated with this composite endpoint in univariate analyses. Kidney function (lower GFR as well as greater proteinuria) was also the strongest determinant of CKD progression in multivariate analyses (multivariate model), along with lower hemoglobin and albumin levels, a history of CVD and elevated hepcidin levels (all p<0.05). The incremental value of hepcidin to the model was described by a (non-significantly, p = 0.12) increased C-statistic of 0.949 ± 0.012 as compared to the model without hepcidin (0.938 ± 0.013).

**Table 4:** Determinants of progression of CKD (Cox proportional hazards analysis).

	univariate	multivariate
hepcidin [10 ng/ml]	**1.179 (1.095; 1.270)**	**1.134 (1.041; 1.235)**
EPO [log(U/L)]	1.463 (0.797; 2.687)	1.13, p = 0.8
age [10 yrs]	**1.330 (1.000; 1.768)**	0.92, p = 0.7
gender, male	**2.100 (1.083; 4.071)**	1.53, p = 0.4
type 2 diabetes	2.432 (0.719; 8.229)	--
GFR [10 ml/min/173m^2^]	**0.431 (0.330; 0.561)**	**0.545 (0.370; 0.802)**
proteinuria [log(mg/day)]	**2.202 (1.740; 2.786)**	**1.542 (1.133; 2.101)**
hemoglobin [g/L]	**0.652 (0.570; 0.746)**	**0.678 (0.503; 0.915)**
CRP [log (mg/dl)]	**1.779 (1.342; 2.358)**	1.28, p = 0.2
albumin [g/dl]	**0.325 (0.218; 0.484)**	**0.301 (0.119; 0.762)**
history of CVD	**2.771 (1.488; 5.162)**	**2.345 (1.035; 5.316)**
hypertension	1.526 (0.596; 3.907)	--
hyperlipidemia	0.894 (0.475; 1.683)	--
ferritin [log(μg/L)]	1.313 (0.918; 1.878)	--

**Legend:** data are hazard ratios (HR) (95% confidence interval, CI), displayed bolded if p<0.05, the multivariate Cox model was built with backwards selection (p_exclusion_>0.05), accounting for variables significant (p<0.05) in univariate association and EPO (HRs and p-values are displayed before variables left the model)

No association of ferritin with *progression of CKD* was observed, neither in univariate/multivariate models, nor when ferritin was forced in the multivariate model. EPO levels were also not related to *progression of CKD* in these analyses, including the interaction with hepcidin or by forcing EPO in the final model. The results were also not altered by forcing in diabetes type and when the analyses were performed on type 2 diabetics only. Results were largely unchanged by excluding iron deficient patients and patients with early stages of CKD (detailed data of sensitivity analyses not shown). Finally, the observed associations were similar in the imputed dataset (S3 Table. Multivariate Cox proportional hazards analysis on imputed dataset, outcome progression of CKD).

Of note, medication with statins or ACE-inhibitors/Angiotensin-Receptor-blockers at baseline was not associated with hepcidin-levels or any of the investigated clinical outcomes in univariate analyses (S4 Table. Univariate linear regression analyses, dependent variable log-hepcidin medication variables; S5 Table. Univariate Cox proportional hazards analyses, outcome mortality, medication variables; S6 Table. Univariate Cox proportional hazards analyses, outcome progression of CKD, medication variables).

## Discussion

In the current cohort study of diabetic patients which were mainly in CKD stages 2 and 3, we found that anemia was common and hepcidin levels were related to endogenous EPO levels and impaired kidney function. These findings were independent from inflammatory processes (e.g. CRP levels) or other clinical conditions (e.g. hypertension, hyperlipidemia). In particular after multivariate adjustment, hepcidin was independently predictive for mortality and *progression of CKD*. We observed an interesting role of EPO in this setting as the interaction of hepcidin and EPO was independently associated with mortality.

Although the pathophysiology of type 1 and type 2 diabetes *per se* is very different, we did not detect any major variation of the associations of hepcidin and EPO and also of both with the investigated outcomes when the type of diabetes was investigated in detail. While based on a considerably small group of patients with type 1 diabetes, these results suggest that the pathophysiology of anemia in diabetic CKD may be similar in large parts, including inflammatory processes as described in ACD.

Hepcidin is the key-hormone of iron metabolism in ACD [14] and inflammatory processes are not only a cause, but also a symptom of iron dysregulation [30–32]. The release of hepcidin-25 leads to internalization and degradation of the iron export channel ferroportin [17]. High hepcidin levels thus result in reduced plasma iron and diminished iron availability. Hepcidin synthesis and release themselves are regulated by changes in iron storage, hypoxia and erythropoiesis [33], and elevated levels of the hormone have been described in association with markers of inflammation (e.g. C-reactive protein, interleukin-6), anemia (e.g. hemoglobin and endogenous EPO) and also with iron status (e.g. ferritin) [18, 34, 35]. We confirmed a strong relationship of hepcidin with ferritin [36] which reflects the pathophysiological mechanism: hepcidin inhibits iron release [18] and thus causes high levels of stored iron, i.e. ferritin. Although a substantial amount of the variability of ferritin is mediated through hepcidin, other factors were independently related to hepcidin, such as GFR and EPO. However, we could not detect any meaningful role of ferritin in predicting mortality or progression of CKD.

Understanding the pathophysiology of ACD in the setting of CKD is important if treatment with iron or ESA needs to be started. It is well known that patients with elevated levels of inflammatory markers need higher doses of ESA to reach certain hemoglobin targets and that these patients are at particularly high risk for mortality [22, 37]. It is still not entirely understood whether it is the use of a higher ESA dose itself or the underlying reasons that necessitate the use of higher ESA doses to achieve target hemoglobin levels that put this group of “non-responders” at particularly “high risk” [22]. Knowing EPO and hepcidin-25 levels [38, 39] might help understanding these processes and to further characterize the group of “high risk” patients.

Data on hepcidin as a risk factor for clinical outcomes in particular in the setting of CKD are sparse. Reports indicated progression of atherosclerotic plaques, and increased risk of CV events and CV mortality [20]. Enhanced oxidative stress caused by iron dysregulation further promotes inflammatory processes and dysregulation of erythropoiesis, such as EPO release and responsiveness of the bone marrow to EPO [5]. *Niihata et al*. found hepcidin being strongly associated with markers of inflammation and independently predictive for the progression of anemia in non-dialysis dependent CKD patients [21]. Chronic (low-grade) inflammation not only is evident in CKD but represents also an important determinant of CVD in this setting [40]. Inflammatory processes have been shown to be predictive for progression of CKD in type 2 diabetics [41, 42], but also for CV-events and for all-cause mortality in patients on hemodialysis. Impairments of iron-regulation, i.e. elevated hepcidin levels, were independently related to CV events and left ventricular mass, but the univariate association of hepcidin with mortality diminished after multivariate adjustment in ESRD-patients of the CONTRAST study [22, 43].

In our cohort of diabetic patients not on RRT we found hepcidin-25 being independently associated with progression of CKD even after adjustment for baseline GFR, proteinuria and other well-known parameters of worse prognosis, such as lower levels of albumin and hemoglobin [44]. Numerically, the predictive ability (C-statistic) was increased by adding hepcidin to the model, but this finding was not significant due to limited statistical power. Analysis of hepcidin regarding its relationship to mortality rendered a potential interplay between hepcidin and endogenous EPO. Elevated levels of both variables indicated an increased mortality risk. Therefore, we hypothesized that if both can be observed in a patient, this would indicate a potentiated risk of mortality. In fact, we found the opposite: the “protective” Hazard Ratio for the interaction term suggested that the worse prognosis was somewhat weakened. Adding hepcidin, EPO, and their interaction also numerically augmented the model’s prognostic information, even after adjustment for age, inflammation (CRP) and proteinuria. Taking into account the limited sample size of our study, thus requiring cautious interpretation and certainly confirmation by future studies, our results suggest that the combination of both variables, hepcidin and EPO, carries important prognostic information.

Hepcidin has demonstrated its role as a biomarker for iron homeostasis [19], but its utility as a predictor of clinical outcome should be explored in more detail, in particular how much incremental prognostic value is carried in addition to established risk factors, such as age, GFR, and anemia. It also needs be investigated, how treatment decisions based on hepcidin measurement may affect clinical outcome, potentially via reducing hepcidin-25 by pharmaceutical interventions which are currently under investigation; these include monoclonal antibodies directed against hepcidin [45] or HIF (hypoxia inducible factor) prolyl hydroxylase inhibitors [46]. Herein, novel treatments focusing on modulation of inflammatory pathways may also yield promising results [47]. However, reduction/suppression of elevated hepcidin-25 or modulation of signal cascades without direct treatment of the potentially underlying conditions that cause elevated hepcidin levels may limit the success of these therapeutic strategies.

Although our study sample is of limited size, the main strengths of the current dataset are a detailed collection of baseline variables and a considerably long observation time of up to 8.6 years. However, we are aware of several limitations of the current results. In both, linear regression, but particularly in the outcome analyses, a large number of explanatory variables were included in the models, thus overfitting of the models and finding associations by chance is surely possible. The limited sample size itself is prone to confounding. We understand our approach as mostly hypothesis generating and our results have to be confirmed by other researchers in independent and larger datasets. Moreover, hepcidin-25 was measured by RIA instead of the gold-standard mass spectrometry and only measurements at baseline were available (without having noted the exact day-time). This is of particular interested, as substantial intra-individual variability of hepcidin-levels was found [48, 49]. Analysis of subsequent hepcidin measurements and analyses of changes of hepcidin-levels over time would surely provide important insights. Finally, we did not collect information on clinical events during follow-up, such as CVD events or stroke, and information on the cause of death was gained from medical records of the patient’s PCP or nephrologist with no assessment by a formal endpoint committee. Therefore, and due to small numbers of outcomes within each category, we did not analyze specific causes of death.

### Conclusions

We found that in diabetic CKD, hepcidin-25 levels were independently associated with endogenous EPO levels and impaired kidney function even after adjustment for markers of inflammation. Hepcidin as the key hormone of iron homeostasis was also predictive for important clinical outcomes, namely *progression of CKD* and mortality. The latter relationship was further described by a significant interaction of hepcidin and EPO. After confirmation by independent studies, our findings suggest that both, hepcidin and EPO may represent important prognostic factors for clinical outcome and may have the potential to further define “high risk” populations in CKD.

## Supporting Information

S1 Methods Methods for imputing missing data. (DOCX)

S1 Table Multivariate linear regression analysis on imputed dataset, dependent variable log-hepcidin. (DOCX)

S2 Table Multivariate Cox proportional hazards analysis on imputed dataset, outcome mortality. (DOCX)

S3 Table Multivariate Cox proportional hazards analysis on imputed dataset, outcome progression of CKD. (DOCX)

S4 Table Univariate linear regression analyses, dependent variable log-hepcidin medication variables. (DOCX)

S5 Table Univariate Cox proportional hazards analyses, outcome mortality, medication variables. (DOCX)

S6 Table Univariate Cox proportional hazards analyses, outcome progression of CKD, medication variables. (DOCX)
