# Supplementary material for: Hepcidin-25 in Diabetic Chronic Kidney Disease Is Predictive for Mortality and Progression to End Stage Renal Disease
Source: PLoS One. 2015 Apr 20;10(4):e0123072. doi: 10.1371/journal.pone.0123072 (PMC4404250; doi:10.1371/journal.pone.0123072)
Supplement: S1 Methods — (DOCX) [file pone.0123072.s001.docx]

**Supplementary materials**

Wagner *et al.* Hepcidin-25 in diabetic chronic kidney disease is predictive for mortality and progression to end stage renal disease

**S1 Methods for imputing missing data**

Missing values (<25% missing) were imputed based on Markov Chain Monte Carlo method (Schafer et al 1997) (SAS procedure proc mi). The following explanatory variables were imputed: EPO (2.8% missing), hemoglobin (3.6% missing), CRP (6.8% missing), albumin (5.6%missing), proteinuria (24.1% missing), and creatinine (0.4% missing). We did not impute data on outcome data (mortality, renal replacement therapy) and hepcidin levels.

*Schafer JL: Analysis of Incomplete Multivariate Data. Chapman and Hall, New York, 1997.*
