# Supplementary material for: Hepcidin-25 in Diabetic Chronic Kidney Disease Is Predictive for Mortality and Progression to End Stage Renal Disease
Source: PLoS One. 2015 Apr 20;10(4):e0123072. doi: 10.1371/journal.pone.0123072 (PMC4404250; doi:10.1371/journal.pone.0123072)
Supplement: S1 Table — (DOCX) [file pone.0123072.s002.docx]

**Supplementary materials**

Wagner *et al.* Hepcidin-25 in diabetic chronic kidney disease is predictive for mortality and progression to end stage renal disease

**S2 Table. Multivariate linear regression analysis on imputed dataset, dependent variable log-hepcidin.**Data are beta-coefficients (95% CI); multivariate linear regression model 2 on imputed dataset; abbreviations: CI, confidence interval; EPO, endogenous erythropoietin; CVD, cardiovascular disease; GFR, glomerular filtration rate; CRP, C-reactive protein.

|  | **imputed dataset**  **N=249** |
| --- | --- |
|  | **β-coefficient (95% CI)** |
| gender, male | 0.219 (0.060; 0.378) |
| EPO [log (U/L)] | -0.018 (-0.027; -0.008) |
| GFR [10 ml/min/1.73m²] | -0.035(-0.066; -0.004) |
| CRP [log(mg/dl)] | 0.063 (-0.001; 0.128), p=0.055 |
| History of CVD | -0.160 (-0.333; 0.013), p=0.070 |
| Hypertension | 0.200 (-0.006; 0.406), p=0.057 |
| Hyperlipidemia | 0.168 (0.009; 0.327) |
