# Supplementary material for: Hepcidin-25 in Diabetic Chronic Kidney Disease Is Predictive for Mortality and Progression to End Stage Renal Disease
Source: PLoS One. 2015 Apr 20;10(4):e0123072. doi: 10.1371/journal.pone.0123072 (PMC4404250; doi:10.1371/journal.pone.0123072)
Supplement: S2 Table — (DOCX) [file pone.0123072.s003.docx]

**Supplementary materials**

Wagner *et al.* Hepcidin-25 in diabetic chronic kidney disease is predictive for mortality and progression to end stage renal disease

**S3 Table. Multivariate Cox proportional hazards analysis on imputed dataset, outcome mortality.**Data are hazard ratios (HR) (95% confidence interval, CI), multivariate Cox model 2 on imputed dataset; abbreviations: EPO, endogenous erythropoietin; CRP, C-reactive protein.

|  | **Imputed dataset** |  |
| --- | --- | --- |
|  | **HR (95% CI)** | **p-value** |
| hepcidin [10 ng/ml] | 1.353 (0.963; 1.901) | 0.080 |
| EPO [log (U/L)] | 2.141 (0.768; 5.964) | 0.144 |
| *Hepcidin * logEPO* | *0.892 (0.787; 1.010)* | *0.070* |
| age [10 yrs] | 1.980 (1.427; 2.747) | <0.001 |
| proteinuria [log(mg/day)] | 1.459 (1.200; 1.774) | <0.001 |
| CRP [log(mg/dl)] | 1.394 (1.069; 1.818) | 0.014 |
