# Supplementary material for: Hepcidin-25 in Diabetic Chronic Kidney Disease Is Predictive for Mortality and Progression to End Stage Renal Disease
Source: PLoS One. 2015 Apr 20;10(4):e0123072. doi: 10.1371/journal.pone.0123072 (PMC4404250; doi:10.1371/journal.pone.0123072)
Supplement: S3 Table — (DOCX) [file pone.0123072.s004.docx]

**Supplementary materials**

Wagner *et al.* Hepcidin-25 in diabetic chronic kidney disease is predictive for mortality and progression to end stage renal disease

**S4 Table. Multivariate Cox proportional hazards analysis on imputed dataset, outcome progression of CKD.**
Data are hazard ratios (HR) (95% confidence interval, CI), multivariate Cox model on imputed dataset; abbreviations: GFR, glomerular filtration rate, CVD, cardiovascular disease.

|  | **Imputed dataset** |  |
| --- | --- | --- |
|  | **HR (95% CI)** | **p-value** |
| hepcidin [10 ng/ml] | 1.098 (1.020; 1.182) | 0.01 |
| GFR [10 ml/min/173m²] | 0.579 (0.434; 0.773) | <0.001 |
| Proteinuria [log(mg/day)] | 1.600 (1.224; 2.090) | <0.001 |
| Hemoglobin [g/L] | 0.676 (0.529; 0.865) | 0.002 |
| Albumin [g/dL] | 0.513 (0.232; 1.135) | 0.09 |
| History of CVD | 2.086 (1.038; 4.191) | 0.04 |
