# Supplementary material for: Hepcidin-25 in Diabetic Chronic Kidney Disease Is Predictive for Mortality and Progression to End Stage Renal Disease
Source: PLoS One. 2015 Apr 20;10(4):e0123072. doi: 10.1371/journal.pone.0123072 (PMC4404250; doi:10.1371/journal.pone.0123072)
Supplement: S4 Table — (DOCX) [file pone.0123072.s005.docx]

**Supplementary materials**

Wagner *et al.* Hepcidin-25 in diabetic chronic kidney disease is predictive for mortality and progression to end stage renal disease

**S5 Table. Univariate linear regression analyses, dependent variable log-hepcidin medication variables.**abbreviations: CI, confidence interval; ACE, angiotensin converting enzyme.

|  | **β-coefficient (95% CI)** |
| --- | --- |
| ACE-inhibitors / Angiotensin II receptor blockers | 0.005 (-0.184; 0.194) |
| Statins | 0.053 (-0.114; 0.220) |
