# Supplementary material for: Hepcidin-25 in Diabetic Chronic Kidney Disease Is Predictive for Mortality and Progression to End Stage Renal Disease
Source: PLoS One. 2015 Apr 20;10(4):e0123072. doi: 10.1371/journal.pone.0123072 (PMC4404250; doi:10.1371/journal.pone.0123072)
Supplement: S6 Table — (DOCX) [file pone.0123072.s007.docx]

**Supplementary materials**

Wagner *et al.* Hepcidin-25 in diabetic chronic kidney disease is predictive for mortality and progression to end stage renal disease

**S7 Table. Univariate Cox proportional hazards analyses, outcome progression of CKD, medication variables.**
Abbreviations: CI, confidence interval; ACE, angiotensin converting enzyme

|  | **Hazard Ratio (95% CI)** |
| --- | --- |
| ACE-inhibitors / Angiotensin II receptor blockers | 0.722 (0.367; 1.423) |
| Statins | 1.463 (0.786; 2.724) |
